# Toll-Like Receptors and Viruses: Induction of Innate Antiviral Immune Responses

**DOI:** 10.2174/1874285800802010049

**Published:** 2008-05-14

**Authors:** Angeliki Xagorari, Katerina Chlichlia

**Affiliations:** 1 Cell and Gene Therapy Laboratory, Dept. of Hematology/BMT, Gen. Hospital G. Papanikolaou, 57010 Exochi, Thes-saloniki, Greece; 2 Dept. Molecular Biology and Genetics, Democritus University of Thrace, Dimitras 19, 68100 Alexandroupolis, Greece

**Keywords:** Toll-like receptors, TLR, innate immunity, virus, RNA helicases, antiviral immune responses.

## Abstract

Induction of antiviral innate immune responses depends on a family of innate immune receptors, the Toll-like receptors (TLR). TLR mediate the antiviral immune responses by recognizing virus infection, activating signaling pathways and inducing the production of antiviral cytokines and chemokines. ssRNA and dsRNA viruses can be recognized by TLR7/8 and TLR3, respectively. TLR receptors are also involved in the recognition of viruses containing genomes rich in CpG DNA motifs as well as envelope glycoproteins. Cytoplasmic recognition of dsRNA by RNA helicases such as RIG-I and MDA5 provides another means of recognizing viral nucleic acid. In order to counteract the innate host immune system viruses evolved mechanisms that block recognition and signaling through pattern recognition receptors, such as TLRs and RNA helicases. Recently, TLR agonists represent a promising approach for the treatment of infectious diseases. This review will focus on the current knowledge of TLR-mediated immune responses to several viral infections.

## INTRODUCTION

Toll-like receptors (TLRs) are crucial in the innate immune response to pathogens, in that they recognize and respond to pathogen associated molecular patterns (PAMPs), which lead to activation of intracellular signaling pathways and altered gene expression. The host innate immune system detects microorganisms and responds to their stimuli mainly through recognition of TLRs [1]. Members of the family of TLRs have emerged as key sensors of innate immunity to viruses recognizing their components such as nucleic acids and envelope glycoproteins. Engagement of TLRs leads to a series of signaling events resulting in the production of type I interferons, inflammatory cytokines and chemokines, and induction of immune responses necessary to eliminate the pathogens [2]. Sensing through TLRs induces maturation of dendritic cells (DCs), thereby initiating adaptive immune responses [3]. Cells also express cytoplasmic RNA helicases that function as an alternative class of pattern-recognition receptors through recognition of double-stranded RNA (dsRNA) produced during virus replication. These two classes of pattern-recognition receptor molecules are expressed in different intracellular compartments and induce type I interferon responses *via *distinct signaling pathways [1,2,4]. TLRs patrol the extracellular and endosomal compartments, while the RNA helicases retinoic acid-inducible gene I (RIG-I) and melanoma differentiation-associated gene 5 (MDA5) survey the cytoplasm for the presence of viral dsRNAs. RNA helicases and the TLR system exert antiviral responses in a tissue- and cell type-specific manner [5].

Viruses are characterized by the complexity of their genomes and they are classified according to the underlying mechanisms of replication (Baltimore classification). The nucleic acid in viral genomes can be either DNA or RNA, positive or negative in polarity, single-stranded or double-stranded, and one continuous (sometimes circular) molecule or a molecule with segmented configuration. Four TLR members seem to play a critical role in recognition of viral nucleic acids [6]: TLR3 recognizes dsRNA (dsRNA constitutes the genome of one class of viruses, but is also generated during the life cycle of many viruses), TLR7 and 8 recognize single-stranded RNA (ssRNA) and TLR9 responds to dsDNA viruses recognizing non-methylated viral CpG-containing DNA. Although the majority of TLRs sense pathogen components on the cell surface, TLR3, TLR7, TLR8 and TLR9 sense nucleic acids in endosomal compartments. Other TLRs are also involved in viral recognition; TLR2 and TLR4 were shown to detect viral components such as envelope glycoproteins [6-9].

**Fig. (1):**
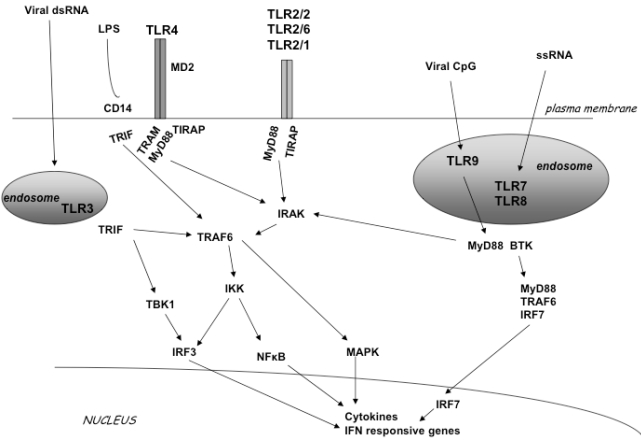
TLR signal transduction pathway. TLR2 can form a homodimer or a heterodimer with TLR1 and TLR6. TLR3, TLR7, TLR8 and TLR9 localize in endosomal compartments. LPS activates TLR4. Each TLR mediates distinctive responses in association with TIR domain-containing adapters (MyD88, TIRAP/MAL, TRIF and TRAM). IRAK and TRAF6 activate NF-κB and induce inflammatory cytokine secre-tion activating the IFN pathway.

Members of the TLR family detect viruses that enter the endosome through endocytosis. This pathway induces production of interferons through several signaling proteins that ultimately lead to the activation of the transcription factors NF-kB, IRF3 and IRF7 [1,10,11] (Fig. **1**). Specifically, upon ligand binding the receptor complex recruits, *via *its cytoplasmic Toll/IL/1 receptor (TIR) domain, the TIR-containing cytosolic adapter protein MyD88, the TIRAP/Mal (appears to function downstream TLR2 and TLR4) and the Toll-IL1 receptor domain-containing adaptor inducing IFN-beta (TRIF, also known as TICAM-1) appears to function downstream of TLR3 and possibly TLR4. The adapter MyD88, in turn, recruits the interleukin 1R-associated kinase (IRAK) complex. IRAK are active kinases dissociating from the receptor-adapter complex upon phosphorylation and associating with TNF receptor-associated factor 6 (TRAF6). TRAF6 then activates at least two distinct signaling pathways leading to the activation of NF-κB and mitogen activated protein kinases (MAPKs), the extracellular signal-regulated kinase (ERK), p38 and the c-Jun N-terminal kinase (JNK) [12]. TRIF appears to be responsible for the induction οf IFN-alpha and IFN-beta gene expression by TLR3 and TLR4 through a MyD88-independent pathway. Two kinases, TBK1 and IKKε, were shown to function downstream of TRIF and upstream of IRF3 (interferon regulatory factor 3), a transcription factor responsible for the induction of IFN genes (Fig. **1**).

Cytoplasmic recognition of dsRNA by RNA helicases such as RIG-I and MDA5 provides another means of recognizing viral nucleic acid [4]. Signaling events downstream of RIG helicases reveal the presence of an adaptor molecule that contains a caspase recruitment domain (CARD). RIG-I activates NF-κB and IRFs through the recently identified adaptor protein MAVS/VISA/Cardif/IPS-1 that resides in the mitochondrial membrane [13]. This adaptor molecule is the sole adaptor in both RIG-I and MDA-5 signaling able to mediate effective responses against a variety of RNA viruses [14].

Type I interferons, IFN-alpha and IFN-beta, are potent antiviral cytokines and modulators of the adaptive immune system. They are induced upon viral infection or by dsRNA and lead to the production of a broad range of antiviral proteins and cytokines. Viruses, in turn, have evolved multiple strategies to counteract the interferon system which would otherwise stop virus growth early in infection [15]. Despite the fact that activation *via *TLR molecules can lead to antiviral innate immune responses, in some cases viruses use these responses to ameliorate infection [16] and to counteract/escape the host immune system [17-19].

## VIRUSES AND TLRs

### Viruses with dsRNA and TLR-3

Toll-like receptor 3 (TLR3) recognizes dsRNA, a universal viral molecular pattern, and thus, is involved in antiviral host immune responses [20]. A synthetic ligand, poly(I:C), can also mediate responses through TLR3. TLR3 has been identified to respond to dsRNA by specifically recognizing purified genomic dsRNA from Lang reovirus and poly(I:C) [20], resulting in the induction of IFN-beta, IL-12, IL-6 and TNF-alpha. However, studies with TLR3-deficient mice provide evidence suggesting that TLR3 does not play a critical role in the host antiviral immune responses to reovirus (dsRNA virus) because susceptibility to infection and generation of T cell immune responses to this virus were equivalent in TLR-deficient and –sufficient mice [20,21]. 

The role of TLR3 was also investigated in rhinoviral infections [22]. Rhinoviruses are the major cause of the common cold and their replication induces expression of TLR3 mRNA and surface protein expression. TLR3 mediates antiviral activity in human bronchial epithelial cells infected with rhinovirus. Blocking of TLR3 during infection impairs the antiviral response, resulting in increased rhinovirus replication [22].

TLR3 plays an important role in the pathogenesis of West Nile virus, a ssRNA flavivirus. This virus replicates through a dsRNA intermediate and causes human disease of variable severity. Infection with West Nile virus leads to a TLR3-dependent inflammatory response that mediates entry and penetration of the virus into the brain causing lethal encephalitis [23]. Viral replication in peripheral tissues triggers inflammation leading to secretion of cytokines such as interferons, IL-6 and TNF-alpha. Signaling through TNFR1 participates in the blood-brain barrier breakdown upon TLR3 stimulation or viral infection with West Nile virus. Thus, TLR3-dependent induction of TNF-alpha facilitates penetration of West Nile virus across the blood-brain barrier and induces neuronal injury [23,24].

Another interesting feature of TLR3 is that it promotes cross-priming to virus-infected cells. It has been proposed that TLR3 may have evolved to permit cross-priming of cytotoxic T cells against viruses that do not directly infect dendritic cells [25].

Evidence to date shows that TLR3 is not universally required for the generation of effective antiviral responses [21,26], proposing a potential role for other pattern recognition receptors. In this regard, RNA helicases represent an alternative major cellular sensor for several viral infections associated with dsRNA. 

### Single-Stranded RNA Viruses and TLR7/TLR8, Other TLRs and RNA Helicases

TLR7 and TLR8 are usually present in the endosomal compartments and sense ssRNA of viruses [27-29] present in the extracellular milieu and engulfed them through the process of endocytosis. Through this exogenous pathway, RNA within internalized virions is detected by TLR7 in endosomes after digestion of the viral envelope and capsid proteins by host cell enzymes. Recently, another pathway had been shown to exist. Plasmacytoid dendritic cells (pDCs) recognize some viruses such as vesicular stomatitis virus (VSV) or Sendai virus through an endogenous pathway, where autophagy performs an analogous function to endocytosis, transferring viral RNA from the cytoplasm to the endosomal compartments containing TLR7 [30,31].

Regardless the route of ssRNA virion internalization, whether by fusion with the plasma membrane (as for VSV-RSV-F or Sendai virus), or by fusion with the endosomal membrane (as for VSV or influenza virus), sensing occurs within the endosome because acidification of the endosomal vacuole is required for an antiviral response [32].

In epithelial cells infected with RSV (a negative ssRNA virus), TLR3 mediates inflammatory cytokine and chemokine production [33]. Mice infected with influenza virus constitutively up-regulate TLR3. Animals deficient in TLR3 show reduced inflammatory mediators and lower number of CD8-positive T lymphocytes in the bronchoalveolar airspace [34]. It is relevant to note that IFN-alpha and ΤΝF-alpha enhance the expression of TLR3, MyD88, TRIF, IRF7 and RIG1 in human lung A549 epithelial cells infected with influenza A virus [35].

Using TLR4-deficient mice it was evident that TLR4 is involved in the innate immunity to respiratory syncytial virus (RSV) through an interaction with the viral envelope fusion protein (F protein) [7,36]. Sendai virus shares many features with RSV including a structurally and functionally similar F protein. Despite these similarities, TLR4 is not involved in host defense against respiratory tract infection with Sendai virus [37].

RNA helicases sense viral infections associated with accumulation of dsRNA in the cytoplasm. Both RIG-I and MDA5 detect dsRNA; however, MDA5 recognizes poly(I:C) and RIG-I detects *in *vitro transcribed dsRNAs [38]. RNA viruses are differentially recognized by these helicases: RIG-I mediates antiviral responses to paramyxoviruses, influenza virus and Japanese encephalitis virus, whereas MDA5 is critical for host antiviral response to picornaviruses [38]. In another study RIG-I was essential for signaling by influenza viruses and human respiratory syncytial virus (RSV) whereas RIG-I and MDA5 were each individually dispensable for signaling triggered by reovirus and dengue virus [39].

Several lines of evidence point to cell-type specificity in the recognition process of infecting viruses [38,40]. For example, activation of signal transduction and induction of cytokine expression by the paramyxovirus Sendai is dependent on virus replication and involves pattern recognition receptors in a cell-type-dependent manner: Cells that are not part of the immune system, such as human embryonic kidney cells, rely entirely on recognition of dsRNA through RIG-1 while cells of myeloid origin, which include macrogphage-like cell lines, utilize TLR7/8 [40]. RIG-I is essential for induction of type I interferons following an infection with ssRNA viruses in fibroblasts and conventional DCs. In contrast, plasmacytoid DCs (pDCs) use the TLR system rather than RIG-I for viral detection [38].

### dsDNA Viruses and TLRs

#### Cytomegalovirus (CMV)

CMV is a dsDNA virus that triggers TLR activation during virus-cell contact and/or entry [8,41]. TLR2 recognize the CMV envelope glycoproteins B and H, leading to innate immune response by activation of NF-kB and induction of inflammatory cytokines [41,42]. Recently, TLR2-deficient mice showed elevated levels of murine CMV (MCMV) in the spleen and liver 4 days post-infection as compared to wild type (wt) C57BL/6 mice and this difference in viral titers was abrogated by *in vivo* depletion of NK cells of TLR2-deficient mice and C57BL/6 mice using anti-NK1.1 antibodies [43]. Therefore, the defect in early antiviral control was associated with reduction of NK cells in the spleen and liver 4 days post-infection. TLR2 was also identified as a receptor which triggers innate immune responses against MCMV [43].

Reactivation of MCMV from latency induced by LPS appears dependent on TLR4 signaling. Specifically, mice receiving intraperitoneal doses of LPS had pulmonary reactivation of latent MCMV 3 weeks following injection with kinetics similar to those of sepsis. When TLR4 signaling was blocked using a monoclonal antibody (MTS510) LPS did not reactivate MCMV [44].

TLR9 mediates the recognition of MCMV as evidenced by experiments using a mutated form of TLR9. A missense mutation in the receptor domain of the Tlr9 gene (TLR9^CpG1^) can be induced by N-ethyl-N-nitrosourea showing unresponsiveness to CpG-containing oligonucleotides. Mice homozygous for the Tlr9^CpG1 ^allele are highly susceptible to an infection with MCMV showing an impaired (infection-induced) secretion of IFN-alpha/beta cytokines and NFκB activation [45].

The TLR9-mediated activation of MyD88 and TLR3-dependent induction of TRIF signaling are activated *in vivo* upon inoculation of MCMV leading to type I interferon production. Notably, neither of the pathways alone - in the absence of the other - offers complete protection against an infection with MCMV, but rather act in an additive or codependent manner [45].

Following an exposure to a CMV antigen, CD123-positive pDCs and CD11c-positive myeloid DCs (mDCs) stimulated with TLR ligands induce autologous memory T cell responses specific for the CMV antigen [46]. Therefore, TLR ligands that specifically target DC subsets can enhance their ability to activate virus-specific T cells and can be used as adjuvants for vaccine or immune modulating therapies.

#### Herpes Simplex Virus Type 1 (HSV1)

The dsDNA virus HSV1 is the major cause of sporadic lethal encephalitis and blindness in humans. Infection with HSV1 induces apoptotic cell death in microglial cells [47].

In addition to the role of TLR2 in infections with CMV, TLR2 is activated in response to Varicella Zoster virus (VZV) [48] and it seems to play significant role in infections with HSV1. Lower levels of cell death are observed in TLR2-/- knock-out cells as compared to wt cells 24 hours post-infection [49].Whereas peritoneal macrophages from TLR2-/- or wt mice are able to produce TNF-alpha in response to an exposure to HSV1 (as opposed to MyD88-/- mice), TLR2-/- mice showed significantly lower levels of monocyte chemoattractant protein 1 (MCP-1) in the brain and reduced mortality compared to wt or TLR4-/- mice [50]. Moreover, an intranasal infection with HSV1 showed that only MyD88-/- knock-out cells were highly susceptible to infection; followed by viral migration to the brain, and severe neuropathological signs of encephalitis and mortality by day 10 post-infection. Summarizing, it seems that innate resistance to HSV1 is mediated by MyD88 and may be activated by multiple TLRs [51]. Additionally, the Affymetrix microarray technology provides evidence that infection of the TLR3-expressing human post-mitotic neuron derivative cell line NT2-N with HSV1 triggers IL6 and IRF1 mRNA production [52]. Thus, human neurons in the absence of glia have an intrinsic mechanism to sense viral infection through TLRs [52].

In response to HSV1 infection, human corneal epithelial cell lines (HCEC, HUCL) - immortalized with telomerase - produce pro-inflammatory cytokines leading to infiltrationin the cornea. The interferons produced enhance antiviral activity in the cornea by sequential activation of TLRs. Specifically, HSV1-infected HCEC cells result in a two-phase activation of NF-κΒ. Concominant with the first peak of activation a number of cytokines are expressed and during the second phase of NF-κB activation TLR7 is induced and TLR3 down-regulated [53].

#### Herpes Simplex Virus Type 2 (HSV2)

CpG motifs, highly present in the genomes of HSV1 and HSV2 as compared to other viral ds genomes, are recognized by TLR9 expressed on pDCs and other types of DCs [54].

Local delivery of CpG oligonucleotides, acting as agonists for TLR9, protects against a lethal challenge with HSV2 by inhibiting virus replication. HEK293 cells transfected with TLR9 provide evidence that the antiviral activity of CpG oligonucleotides is mediated through TLR9 [55]. Administration of poly(I:C) protected mice showing increased survival of mice even with a challenge infection with a 10-fold-higher HSV2 load [56].In contrast, treatment with TLR4 or TLR2 ligands does not lead to a protective effect against a challenge infection with intravaginal HSV2 [57].

Vaginal infection with tk-HSV2, a recombinant HSV2 expressing thymidine kinase, results in a rapid recruitment of CD11b-positive DCs to the submucosa with their subsequent appearance in the lymph nodes presenting viral peptides to CD4-positive T cells [58]. DCs require signals from stromal cells - which are mediated through MyD88 - in order to promote generation of Th1 cells [59].

#### Adenovirus (AdV)

Adenovirus (AdV) is a dsDNA virus causing respiratory, ocular and gastrointestinal illness in humans. AdV activates innate immunity by its DNA through TLR–dependent and –independent pathways in a cell-type specific fashion [60,61]. The recognition of AdV by pDCs is reported to be mediated by TLR9 and is dependent on MyD88. In contrast, recognition by non-pDCs is TLR-independent through cytosolic sensing of adenoviral DNA. MyD88-/- knock-out mice confirmed that the AdV-induced dysregulation of functionally related gene clusters is significantly dependent on this adaptor molecule which plays a significant role as an amplifier and regulator of adenoviral immunity *in vivo *[62].

Recognition of AdV by TLR9 requires the adenoviral DNA. Efficient endosomal acidification of oligonucleotides that trigger TLR9 attenuates induction of IFN-alpha gene expression. Cell lines expressing TLR9, permissive to infection by both adenoviral serotypes utilizing the Coxsackievirus-AdV Receptor (CAR) and serotypes utilizing CD46, show a preferential induction of TLR9-mediated events by AdVs utilizing CD46 for their entry [63]. Therefore, infectivity alone is not sufficient for TLR9 activation but seems to be regulated by the specific receptor entry pathway [63].

#### Human Papillomavirus (HPV)

Human papillomavirus (HPV) is another dsDNA virus; many data strongly suggest that HPV infection is causative agent of cervical cancer [64]. Infection with HPV 16L1 virus-like particles (VLPs) provides immunity by activating DCs and a potent neutralizing IgG response, which requires MyD88-dependent signaling. IFN-alpha/beta as well as numerous proinflammatory cytokines and chemokines are up-regulated in response to an infection with HPV16 VLPs [65]. Bone marrow-derived DCs deficient in MyD88 failed to up-regulate IL12, IFN-alpha and IFN-gamma in response to HPV16VLPs. Moreover, Th1-biased immune responses are impaired in mice deficient in MyD88 and IFN-alpha/beta receptor. These observations implicate TLRs to have a central role in immune recognition of HPV16L1 VLPs [66]. The TLR7 agonist, imiquimod is already marketed for papillomavirus disease [67].

#### Vaccinia Virus and TLRs

Innate immune recognition of vaccinia virus is mediated by TLR2 and MyD88 and requires TLR-independent production of IFN-beta [68]. Both TLR-dependent and –independent pathways are required for the activation of innate and adaptive immunity to vaccinia virus *in vivo* [68].

Vaccinia virus has been extensively used as a vaccine vehicle in clinical application in several studies. Previous studies suggested that the unique potency of vaccines based on vaccinia virus lies in its effective activation of the innate immune system [68,69]. Using microarrays it was shown that TLR3 is specifically induced upon infection of immature human monocyte-derived DCs with the attenuated poxvirus vector MVA [70].

#### Epstein-Barr Virus (EBV) and TLRs

Epstein-Barr virus (EBV) infection of primary B cells causes B cell activation and proliferation. TLR signaling has been reported to provide a third B cell activation stimulus. EBV up-regulates the expression of TLR7 and downregulates the expression of TLR9 in naïve B cells [71]. IRF-5 is induced following EBV infection and B cell proliferation assays provide evidence that EBV modulates TLR7 signaling [71]. Upon EBV infection a novel splice variant of IRF-5 transcripts is induced acting as a dominant negative form. EBV therefore initially uses TLR7 signaling to enhance B cell proliferation and subsequently modifies the pathway to regulate IRF-5 activity [71]. TLR2 is also shown to play an important role in immune responses directed against EBV infection. Infectious and UV-inactivated EBV virions are demonstrated to lead to the activation of NF-κB through TLR2 [9]. In addition, EBV infection of primary human monocytes induces the release of the chemokine monocyte chemotactic protein 1 (MCP-1), and small interfering RNAs targeting TLR2 significantly reduce the chemokine response to EBV [9].

#### Retroviruses and TLRs

The internalization of the retrovirus human immunodeficiency virus type 1 (HIV-1) is mediated by interactions of the viral envelope with the host CD4 receptor. Viral RNA present in endosomal compartments and recognized by TLR7 [72] is the critical factor that stimulates pDCs and induces IFN-alpha secretion. Uridine-rich ssRNA derived from the HIV-1 long terminal repeat were shown to serve as potent activators of NK cells *via *TLR7/8 [73]. HIV-1-derived TLR ligands can contribute to the immune activation of NK cells and may play an important role in HIV-1-associated immunopathogenesis as well as NK cell dysfunction observed during acute and chronic viremic HIV-1 infection [73]. Constitutive association of MyD88 with IRAK1 is evident in Human T cell leukaemia virus type 1(HTLV-1)-transformed T cells; HTLV-1 Tax enhances TLR expression and synergistically activates NF-κB through wt MyD88 [74]. HTLV-1 has also evolved a protein that interferes with TLR4 signaling. HTLV-1 p30 interacts with PU.1 and inhibits its DNA binding and transcription activity resulting in the down-regulation of TLR4 expression from the cell surface [75]. Expression of p30 hampers the release of pro-inflammatory cytokines and stimulates release of anti-inflammatory cytokines like IL-10 following stimulation of TLR4 in human macrophages [75]. This novel function of p30 may explain the reduced activation of adaptive immunity in adult T-cell leukemic (ATL) patients. TLR4 also induces maturation of bone marrow-derived dendritic cells and the up-regulation of the MMTV entry receptor (CD71) on these cells [76].

### Hepatitis C Virus (HCV) and TLRs

Hepatitis C virus (HCV) is an enveloped, ssRNA virus, able to establish chronic infections. Pathogens are common in HCV-infected patients and can be recognized by TLRs, which are upregulated in monocytes and T cells [77,78].

DCs sense virus infections *via *TLRs and RIG pathways and produce large amounts of type I IFNs and inflammatory cytokines [79]. Reduced expression of TLR2 on immature DCs from HCV-infected patients compared to the control group results in a lesser ability to stimulate T cell proliferation [80]. Dysfunctions of HCV-DCs have been reported by several other investigators. Hepatitis C virus purified from serum of chronically infected patients in the form of lipo-viro-particles (LVP, triglyceride-rich lipoprotein like particles containing viral RNA and proteins) interacts with TLR4 and activates DCs to mature and to induce through ERK and p38-dependent mechanisms a Th2-biased phenotype instead of a Th1 [81]. Th2 cells produce IL4, IL5, IL6, IL9, IL10 and IL13 cytokines that promote B cell expansion and downregulate Th1 cells [79].

Plasmacytoid DCs (pDCs) are known to secrete large amounts of IFN-alpha in response to TLR activation and are capable of activating naïve T cells. In HCV-infected patients pDCs exhibit reduced responsiveness to TLR ligation (R848 stimulation), accompanied by reduced expression of the marker HLA-DR and the cytokine IFN-alpha, and impaired activation of naïve CD4-positive T cells [82]. In myeloid DCs from HCV-infected patients, the levels of TLR/RIG-I-mediated IFN-beta or TNF-alpha induction are lower than those in uninfected donor despite the high expression of TLR2, TLR4 and RIG-1, meaning that the signal transduction is impaired in HCV-infected cells [83].

There are many evidences showing that HCV components can bind TLRs and activate their signaling pathway or block TLR function by interfering with intracellular intermediates [78,84-88]. Several reports have shown an immuno-modulatory role of the HCV core protein [84]. The HCV core protein can associate with the cell surface receptor of the recognition component C1, gC1q receptor (gC1qR) on human monocyte-derived DCs and inhibit TLR4-induced IL12 production without affecting the production of other TLR-stimulated cytokines [84,89]. Likewise, incubation of mononuclear cells with the HCV core protein results in up-regulation of TLR2 expression and suppression of TLR4 and TLR7 in patients and controls [78]. TLR2 may use TLR1 and TLR6 co receptors for TLR2 activation of macrophages and innate immunity in humans and mice [85, 90]. Studies with mouse macrophage cell lines stably expressing the HCV non structural proteins (NS3, NS3/4A, NS4B or NS5A) showed inhibition of TLR2, TLR4, TLR7 and TLR9 signaling pathways [87]. Analysis of the effect of the entire or parts of the HCV open reading frame (core-NS3, NS5-NS5B) in a liver derived cell line, HepG2, has shown that TLR3 expression is suppressed in the transfectants expressing the entire HCV open reading frame whereas it is elevated in the transfectant expressing core-NS3 [78]. Thus, suppressed expression of TLR3 may be responsible for the persistence of the virus in chronic HCV infection.

### Hepatitis B Virus (HBV) and TLR

Hepatitis B virus (HBV) is a dsDNA virus, which may cause acute and chronic infections. The HBV surface antigen (HBsAg) is most frequently used to screen for the presence of this viral infection. TLRs are involved in controlling HBV infection. Intravenous injection of transgenic mice with TLR2, TLR3, TLR4, TLR5, TLR7 and TLR9 showed that all the ligands except TLR2 inhibited HBV replication in the liver non-cytopathically within 24 hours in an IFN-alpha/beta-dependent manner [91]. Other studies reported that TLR2 is reduced in HBV-infected mononuclear cells from peripheral blood, whereas TLR4 expression was higher as compared to other TLRs [92].

Analysing the effect of the 3’CCACCA motif of tRNA ^Ala^ (UCG) on the immune response of the hepatitis B antigen (HBsAg) in BALB/c mice showed that this motif increased Th1 and CTL immune responses [100]. Notably, this motif can be recognized by TLR3. In this regard, deletion of the 3’CCACCA sequence of tRNA ^Ala ^decreased the recognition through TLR3 [93].

### Viral Evasion of the Innate Host Immune System

In order to establish successful infections viruses need to counteract the innate host immune defences evolving mechanisms that block recognition and signaling through pattern recognition receptors, such as TLRs and RNA helicases. The involvement of viral pattern recognition receptors and possible immune evasion mechanisms are listed in Table **1**. For example, the V proteins of paramyxoviruses bind to MDA5, thereby inhibiting the activation of the IFN-beta promoter [94]. The P protein of measles virus suppresses TLR signaling through up-regulation of the ubiquitin-modifying enzyme A20 [95]. NS1 protein of influenza A virus antagonizes the host antiviral response by inhibiting the function of RIG-I. The expression of NS1 downregulates production of IFN-beta induced by RIG-I agonists, and ectopic expression of RIG-I inhibits the replication of influenza A virus [96]. The adenoviral E3 protein 14.7K inhibits antiviral immunity and inflammation by blocking the activity of NF-κB following signaling through TLR and TNFR [97]. 

Studies on the mechanism of HCV evasion provided strong evidence for a virus-specific proteolysis of TRIF, an adaptor protein, which links TLR3 and kinases mediating activation of IRF3 and NF-κB. The cleavage is mediated by the HCV protease NS3/4A, responsible for inhibition of poly(I:C)-activated signaling through the TLR3 pathway [98]. Overexpression of IKKε is able to inhibit positive and negative replicative strands of the HCV replicon [99]. The RIG-1 signaling pathway is mediated by the antiviral mitochondrial protein MAVS/VISA/Cardif/IPS-1 which activates NF-κB and IRFs. HCV employs the viral protease NS3/4A to cleave MAVS at Cys-508 resulting in the dislocation of the N-terminal fragment of MAVS from the mitochondria [88].

In order to counteract the host innate immunity vaccinia virus encodes immunomodulatory proteins that antagonize important components of the host antiviral defense [100]. This virus encodes sequences similar to the TIR domain that are able to interfere with NF-κB signaling. The vaccinia virus protein A52R was shown to intracellularly block the activation of NF-κB induced by multiple TLRs, including TLR3 [101]. This mechanism of disabling TLR signaling by association of A52R with IRAK2 and TRAF6 is used by vaccinia virus in order to suppress host immunity [101]. Moreover, although A52R inhibits TLR-mediated NF-κB activation it can simultaneously activate MAP kinases and was shown to mediate enhancement of TLR-induced IL-10 [102]. The viral protein A46R, having a distinct function from A52R, targets multiple TIR adaptor molecules, like MyD88 and TRIF, and contributes to virulence. A recombinant vaccinia virus lacking A52R was attenuated in a murine intranasal model, demonstrating importance of this protein to the virulence [103]. Another poxvirus protein, N1L, was also shown to inhibit NF-κB and IRF3-mediated signaling through TLRs [104].

Collectively, viral evasion of innate immune responses is achieved by expression of viral proteins that inhibit important molecules in the signalling cascades triggered by TLRs and RNA helicases.

### TLR Agonists in Viral Infections

TLRs induce protective immune response against infections. Novel compounds designed to act as potent agonists can be used as therapeutics. The TLR3 agonist poly(I:C) has demonstrated antiviral effects in animals [105]. Oligonucleotides that act as TLR9 agonists can lead to cellular activation and cytokine production influencing immune response against viruses [106].

Recently, the HIV-1 gag protein has been conjugated to the TLR7/8 agonist and shown to improve the magnitude and quality of T cell responses in non-human primates [107]. The TLR7 agonist R-848 can enhance HBsAg-specific humoral and cellular immune responses and together with CpG oligonucleotides may be used as adjuvants for therapeutic and prophylactic HBV vaccine formulations [108]. Αnother TLR7 agonist, imiquimod, has been successfully used in the treatment of HPV [106] and HSV infections [105]. The synthetic imidazoquinoline compounds as TLR8 agonists exhibit immunostimulatory activity [109]. Currently, a number of TLR agonists are under investigation and in clinical trials, such as the anti-HIV TLR3 agonist poly(I:C_12_U), the TLR7 agonist imiquimod, the TLR7/8 agonist resiquimod against HSV and HCV, the TLR7 agonist isatoribine, ANA975 and the TLR9 agonist CPG10101 against HCV [105].

Extensive knowledge of TLR-dependent viral recognition may lead to the generation of new adjuvants and antiviral agents. Targeting specific DC subsets with TLR ligands can enhance their ability to activate virus-specific T cells, providing information for the rational design of TLR ligands as adjuvants or immuno-modulators [110].

## CONCLUSIONS/PERSPECTIVES

Members of the TLR family are important pathogen-recognition receptors and provided a breakthrough in the field of microbial pathogenesis and human immunology over the recent years. TLRs are present at the cell surface such as TLR2 and TLR4, or in endosomal compartments (TLR3, TLR7, TLR8). In the cytoplasm RNA helicases represent another class of pattern recognition receptors that respond to dsRNA. Viral genomes are recognized by different classes of TLRs; ssRNA viruses are recognized mainly by TLR7 and TLR8, TLR3 responds to dsRNA but also in some cases to ssRNA and TLR9 to dsDNA viruses. Consequently, TLRs activate signaling pathways leading to the induction of many immuno-active cytokines and chemokines. The nucleic acids in the viral genomes or envelope glycoproteins or synthetic molecules mimicking microbial structures can be detected by TLRs triggering antiviral defence mechanisms. Other cytosolic receptors, like the cytosolic sensor of dsDNA DAI (DLM-1/ZBP1) [111], may also prove to have important roles in recognition and antiviral immune responses.

While TLR activation triggers antiviral immune defences, excessive TLR activation induced by viruses may have detrimental effects for the host. It is necessary to elucidate the harmful effects resulting from excessive TLR activation in viral infections as in the case of West Nile Virus [23].

The differential and redundant roles of different TLRs and RNA helicases in viral recognition and innate immune signaling may reflect unique and shared biological properties of certain viruses. The differential triggering of pattern recognition receptors and control of their gene expression [39] as well as their tissue and cell-type specificity may impact pathogenesis and infection.

## Figures and Tables

**Table 1.** TLRs (or RNA Helicases) in Viral Infections and Possible Evasion Mechanisms of TLR-Mediated Antiviral Immunity

Class*	Virus family	Virus	Type of nucleic acid	TLR (or RNA helicases)	Possible immune evasion mechanism
I	Herpesviridae	Herpes Simplex Virus	ds DNA, enveloped	TLR2 [49,50], TLR3 [52], ΤLR7 [53], TLR9 [54]	
		Varicella Zoster Virus	ds DNA, enveloped	TLR2 [48]	
		Cytomegalovirus	ds DNA, enveloped	TLR2/CD14 [42], TLR3 [20,45], TLR9 [45]	
		Epstein Barr Virus	ds DNA, enveloped	TLR2 [9], TLR7 [71]	EBV manipulates TLR7 signaling [71]
	Poxviridae	Vaccinia Virus	ds DNA, complex coats	TLR2 [68]	N1L inhibits NFkB and IRF3 signaling [104]
					A52R and A46R inhibit TIR signaling and NFkB activation [100,101,103]
	Adenoviridae	Adenovirus	ds DNA, naked	TLR9 [61,63]	
	Papovaviridae	Papillomavirus	ds circular DNA, naked	MyD88 [66], TLR7 [67]	
II	Parvoviridae	Parvovirus (Adeno-associated)	ss DNA, naked	TLR9 [112]	
III	Reoviridae	Reovirus	ds RNA, segm., naked	TLR3 [20], RIG-I, MDA5 [39]	
IV	Picornaviridae	Rhinovirus	ss RNA	TLR3 [22]	
		Encephalomyocarditis virus	ss RNA	MDA5 [38]	
		Hepatitis A virus	ss RNA	MDA5 [38]	3ABC cleavage of MAVS [113]
	Flaviviridae	Hepatitis C virus	ss RNA, enveloped	TLR2 [80], TLR3[98], TLR4 [81], TLR7 [80][77,78],	NS3/4A cleavage of TRIF [98] and MAVS [88]
				TLR2/1, TLR2/6 [85], RIG-I [99]	NS5A interacts with MyD88 and impairs TLR signaling [87]
		West Nile virus	ss RNA, enveloped	TLR3 (TNFa) [23]	
		Japanese encephalitis virus	ss RNA, enveloped	RIG-I [38]	
		Dengue virus	ss RNA, enveloped	RIG-I, MDA5 [39]	
V	Orthomyxoviridae	Influenza virus	ss RNA, segm, enveloped	TLR3 [33], TLR7 [27,29], RIG-1 [35,38,39]	NS1 protein inhibits function of RIG-I [96]
	Paramyxoviridae	Measles virus	ss RNA, enveloped	TLR2 [115], TLR4 [95]	P protein suppresses TLR signaling through upregulation of A20 [95]
		Respiratory syncytial virus	ss RNA, enveloped	TLR4 [7,36], TLR3 [33], TLR7/MyD88 [116],RIG-I [39]	
		Sendai virus	ss RNA	TLR7/8 [40], RIG-1 [38]	V protein binds to MDA5 and inhibits its activity [94]
		Newcastle Disease virus	ss RNA	RIG-1 [38]	
	Rhabdoviridae	Vesicular Stomatitis virus	ss RNA	TLR3 [20], TLR7/MyD88 [29], RIG-1 [38]	
	Arenaviridae	Lymphocytic Choriomeningitis Virus LCMV	ss RNA, 2 RNAs	TLR3 [20]	
VI	Retroviridae	HIV	ss RNA, enveloped	TLR7/8 [72,73]	
		HTLV	ss RNA, enveloped	MyD88 [74], TLR4 [75]	p30 interferes with TLR4 signaling [75]
		MMTV	ss RNA, enveloped	TLR4 [76]	
VII	Hepadnaviridae	Hepatitis B virus	ds circular	TLR2 [91,92], TLR3 [93], TLR4, TLR5, TLR7 [91]	

^*^according to Baltimore classification of viruses.

## ABBREVIATIONS

AdV: adenovirus
BTK: Bruton’s tyrosine kinase
CARD: caspase recruitment domain
CMV: cytomegalovirus
DAI: DNA-dependent activator of IFN-regulatory factors
DCs: dendritic cells
ds: double-stranded
EBV: Epstein-Barr virus
HCV: Hepatitis C virus
HIV: human immunodeficiency virus
HSV: Herpes simplex virus
HTLV: Human T cell leukaemia virus
IFN: interferon
IKK: I-κb kinase
IPS-1: interferon-beta promoter stimulator 1
IRAK: IL-1 receptor associated kinase
IRF: interferon regulatory factor
LCMV: Lymphocytic choriomeningitis virus
Mal: MyD88-adaptor like
IPS-1: interferon-beta promoter stimulator 1
MAVS: mitochondrial antiviral signalling
MCMV: mouse cytomegalovirus
MDA5: melanoma differentiation-associated gene 5
mDC: myeloid DC
MMTV: mouse mammary tumor virus
MVA: Modified vaccinia virus Ankara
MyD88: myeloid differentiation primary response gene 88
NF-κB: nuclear factor kappa B
PAMP: pathogen-associated molecular pattern
pDC: plasmacytoid DC
RIG-I: retinoic acid-inducible gene I
ss: single-stranded
RSV: Respiratory syncytial virus
SeV: Sendai virus
TICAM: Toll-interleukin 1 receptor domain-containing adaptor molecule
TIR: Toll-interleukin 1 receptor domain
TIRAP: TIR-containing adaptor protein
TNF: Tumor necrosis factor
TRAF: tumor necrosis factor receptor-associated factor
TRIF: TIR-domain containing adaptor inducing TNF-beta
VISA: virus-induced signalling adaptor
VSV: vesicular stomatitis virus
